# Reference-free compression of high throughput sequencing data with a probabilistic de Bruijn graph

**DOI:** 10.1186/s12859-015-0709-7

**Published:** 2015-09-14

**Authors:** Gaëtan Benoit, Claire Lemaitre, Dominique Lavenier, Erwan Drezen, Thibault Dayris, Raluca Uricaru, Guillaume Rizk

**Affiliations:** 1 INRIA/IRISA/GenScale, Campus de Beaulieu, Rennes, 35042 France; 2 University of Bordeaux, CNRS/LaBRI, Talence, F-33405 France; 3 University of Bordeaux, CBiB, Bordeaux, F-33000 France

**Keywords:** Compression, *de Bruijn Graph*, NGS, Bloom filter

## Abstract

**Background:**

Data volumes generated by next-generation sequencing (NGS) technologies is now a major concern for both data storage and transmission. This triggered the need for more efficient methods than general purpose compression tools, such as the widely used gzip method.

**Results:**

We present a novel reference-free method meant to compress data issued from high throughput sequencing technologies. Our approach, implemented in the software Leon, employs techniques derived from existing assembly principles. The method is based on a reference probabilistic *de Bruijn Graph*, built *de novo* from the set of reads and stored in a Bloom filter. Each read is encoded as a path in this graph, by memorizing an anchoring kmer and a list of bifurcations. The same probabilistic *de Bruijn Graph* is used to perform a lossy transformation of the quality scores, which allows to obtain higher compression rates without losing pertinent information for downstream analyses.

**Conclusions:**

Leon was run on various real sequencing datasets (whole genome, exome, RNA-seq or metagenomics). In all cases, LEON showed higher overall compression ratios than state-of-the-art compression software. On a *C. elegans* whole genome sequencing dataset, LEON divided the original file size by more than 20.

Leon is an open source software, distributed under GNU affero GPL License, available for download at http://gatb.inria.fr/software/leon/.

**Electronic supplementary material:**

The online version of this article (doi:10.1186/s12859-015-0709-7) contains supplementary material, which is available to authorized users.

## Background

It is well known that data volumes produced by next-generation sequencing are a major issue. The size of the Sequence Read Archive, hosting a major part of the sequence data generated world wide, is growing very fast and now contains 3.5 petabases (http://www.ncbi.nlm.nih.gov/Traces/sra/) of DNA and RNA [1]. This is an issue for both data storage and transmission, hampering collaboration between teams and long-term storage of data needed for the reproducibility of published results. Raw reads are stored in ASCII-based text files, in FASTA or FASTQ formats, containing for each read entry a read ID, a string for the sequence itself and, for the FASTQ files, a string of quality scores encoding a per base estimation of accuracy. Such files are usually compressed with the general purpose compression tool GZIP (www.gzip.org, Jean-Loup Gailly and Mark Adler), which is fast and largely accepted but does not exploit specificities of sequencing data

Compression of sequencing data can be divided into three distinct problems: compression of read IDs, of base sequence and of quality scores. For the compression of read IDs, standard methods are perfectly suited, since read IDs are usually highly similar from one read to another. Compression of DNA sequences and quality scores on the other hand, are two very different problems. The former displays high redundancy across reads when depth of sequencing is high, but spread over the whole file, and must be lossless, whereas the latter displays a highly noisy signal on a larger alphabet size, and lossy compression may be appropriate. Here we present a software for the compression of FASTA and FASTQ files, including read IDs, DNA sequences and quality scores.

*Sequence compression* techniques fall into two categories: reference-based methods, such as QUIP, CRAM, PATHENC and FASTQZ, exploit similarities between reads and a reference genome [2–5], whereas *de novo* compression schemes in FQZCOMP, SCALCE, FASTQZ, DSRC, ORCOM, BEETL, MINCE exploit similarities between reads themselves [5–10]. Reference based methods usually map reads to the genome and then only store information needed to rebuild reads: genome position and differences. While efficient, such methods require a time-consuming mapping phase to the genome, and are not applicable when no close reference is known. Moreover, the reference genome is also needed for de-compressing the data, which could lead to data loss if the reference has been lost or modified.Most *de novo* methods either (i) use a context-model to predict bases according to their context, followed by an arithmetic encoder (FASTQZ, FQZCOMP, DSRC), or (ii) re-order reads to maximize similarities between consecutive reads and therefore boost compression of generic compression methods (SCALCE, ORCOM, MINCE).

FASTQZ and FQZCOMP are improvements of generic text-compression methods. For simple genomes, the context model is able to learn the underlying genome and produces good results. DSRC uses similar techniques, but is additionally tailored for high-speed compression and decompression. PATHENC also uses a context model followed by arithmetic coding, with two refinements [4]. First, the context-model is initialized with a reference genome. Secondly, the starts of the reads are encoded separately, as a set of kmers compactly encoded with a bit tree, which also requires to reorder reads. While they conceptually make a connection with paths in a graph, their method does not use a de Bruijn graph.

As far as we know, tools that achieve the best sequence compression ratios are currently read re-ordering methods.BEETL and BEETL-FASTQ use the Burrows-Wheeler transform of the read set to achieve compression [9, 11]. However, the method seems to be more suitable as a searchable compressed archive than a full compression/decompression tool of fastq files (see BEETL-FASTQ Readme). ORCOM uses minimizers, an increasingly used method in various NGS algorithms, to quickly re-order the read set into bins of reads of high similarity [8]. This is a very efficient method regarding sequence compression ratio and execution speed. Their method is currently limited to sequence compression, header and quality streams are discarded. MINCE exploits the same paradigm, it also re-order reads into buckets of similar reads based on minimizers. Reads are then transformed to avoid redundant coding of the minimizer among a bucket, and compressed by a general-purpose compressor. Header and quality streams are discarded. However, when reordering reads, one should pay special attention to keeping read pairing information. Indeed losing such information would make it impossible to use down-stream NGS analysis requiring paired-reads. In this sense, methods that reorder reads without dealing with the pairing information cannot be considered as lossless, and cannot be directly compared to other methods. MINCE addresses this issue by concatenating paired reads together before compression, and splitting them after decompression. SCALCE also has an option to handle paired-reads correctly, but activating this option significantly degrades the compression ratio.

Lastly, QUIP (in its reference-free mode) uses a different approach, based on methods tailored for NGS analysis. Sequence assembly algorithms building a reference genome as a set of contigs are used, followed by a reference-based approach [2]. This method is highly dependent on the quality of the generated contigs, and is out-performed in a recent compression competition [5].

*Quality score compression* techniques are divided between *lossless* methods, where decompressed data is guaranteed to match the original values, and *lossy* approaches, trading loss in fidelity of reproduction for higher compression rates.

It has been observed that quality values are generally correlated to their position in the read and to the nearby quality values. Many *lossless* methods exploit this through the use of context-models followed by arithmetic coding (DSRC, FASTQZ, FQZCOMP). Other *lossless* approaches transform quality scores to values that can be coded using fewer bits, *i.e.* gap translating, min shifting and frequency ordering [12].

Classic *lossy* approaches consist in reducing the range of possible values, making further compression easier. The general idea is to divide the initial spectrum of scores into a lower number of bins. FASTQZ and Wan et al. present variations on this scheme [5, 12]. FQZCOMP and LIBCSAM methods smooth qualities within a block, ensuring that the difference from the original value is no more than a given threshold [5, 13].

Other *lossy* quality scores compression approaches use the information contained in the DNA sequence to make smart modifications of the quality scores by smoothing unimportant quality values, in order to reduce the entropy of the quality set. Janin et al. assume that if a given nucleotide can be completely predicted by its context, then its corresponding quality value becomes unimportant and can even be discarded [14]. This is achieved through a time and memory-consuming Burrows-Wheeler transform (BWT) of the read set and a longest common prefix (LCP) array. The method RQS [15] exploits a similar idea but, instead of computing the BWT, compute a dictionary of frequently occurring kmers and then identify kmers within small Hamming distance of frequent ones. Positions corresponding to differences from such frequent kmers are assumed to be SNPs or sequencing errors and their quality values are preserved, while the other quality scores are smoothed. Surprisingly, RQS improves SNP-calling accuracy on a gold-standard dataset. However, their method scales to large read sets only if the dictionary is constructed over a sample of the original data set, and the effect of such sampling is not clearly measured.

In this paper, we introduce LEON, a novel *de novo* method for lossless sequence compression and lossy quality compression using methods derived from assembly principles. However, instead of building a reference as a set of sequences, the reference is represented as a probabilistic *de Bruijn Graph*. Read order is preserved and each read is represented by a kmer anchor and a list of bifurcation choices, enough to re-build it from the graph. The same data structure is used for quality compression. Nucleotides covered by a sufficient number of highly frequent kmers are assumed to be error-free and have their quality smoothed to an arbitrarily chosen value. The quality stream is then compressed with the zlib library.

LEON was run on various real sequencing datasets to evaluate the impact on compression ratio of numerous dataset features, such as the size and complexity of the target genome, the sequencing depth, the sequencing technology. In all cases, LEON showed higher overall compression ratios than state-of-the-art compression software and was at least 2 times faster than the generic tool GZIP, with at least 5 times better compression ratios. For instance, on a *C. elegans* whole genome sequencing dataset,the original file size is divided by more than 20, and in a large human dataset case, the file size was reduced from 733 GB to 47 GB. Several types of sequenced samples (whole genome, exome, RNA-seq or metagenomics) were also tested, demonstrating the robustness of LEON. Finally, the effects of the lossy transformation of quality scores was evaluated on a SNP calling analysis and showed an improvement in the prediction accuracy.

## Methods

### Overview

Although our compression approach does not rely on a reference genome, it bears some similarities with reference-based approaches. As we do not dispose of any external data, the first step of our approach is to build *de novo* a reference from the reads and then, similarly to reference-based approaches, to record each read as a position and a list of differences with respect to this reference. However, the major difference lies in the data structure hosting the reference: instead of a sequence or a set of sequences, a *de Bruijn Graph* is built, whose basic pieces of information are *kmers*, i.e. words of size *k*. This data structure, commonly used for *de novo* assembly of short reads, has the advantage of representing most of the DNA information contained in the reads while dumping the redundancy due to sequencing coverage.

Since the *de Bruijn Graph* must be stored in the compressed file to reconstruct the reads, one important issue is its size. To tackle this issue, our method relies first on a good parameterization of the *de Bruijn Graph* and secondly on its implementation as a *probabilistic* data structure. The parameters are set so that the structure stores most of the important information, that is the most redundant one, while discarding the small differences, such as sequencing errors. Our implementation of the *de Bruijn Graph* is based on bloom filters [16]. Although not exact, this is very efficient to store such large data structures in the main memory and then in the compressed files.

**Fig. 1:**
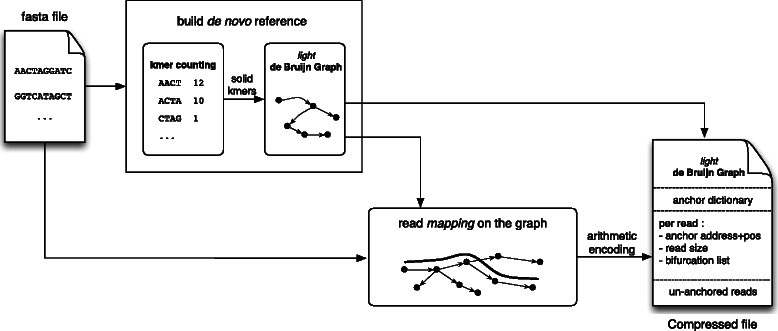
LEON method overview. First, a *de Bruijn Graph* is constructed from the reads: kmers are counted, then abundant enough kmers are inserted into a bloom filter representing a probabilistic *de Bruijn Graph*. Reads are then mapped to this graph, and the necessary information required to rebuild the reads from the graph is stored in the compressed file: an anchoring kmer and a list of bifurcations

Figure 1 shows an overview of the method implemented in the LEON software. First, kmers are counted and only those abundant enough are inserted into a bloom filter representing the *de Bruijn Graph*. Each read is encoded by first finding its best anchoring kmer, then a walk through the graph starting from this anchor node is performed to construct the list of bifurcations followed when mapping the read to the graph. Finally, the compressed file contains the *de Bruijn Graph* and, for each read, its anchoring kmer and a list of bifurcations encoded with an order 0 arithmetic encoder.

### Building the reference as a *de Bruijn Graph*

A *de Bruijn Graph* is a directed graph where each node is a word of length *k*, called a kmer. An edge is present from node *a* to node *b* if the *k*−1 suffix of node *a* is exactly the *k*−1 prefix of node *b*. A *de Bruijn Graph* can be built from a set of reads by cutting each read in overlapping kmers. Each read of size *l* is then a path of *l*−*k*+1 nodes in the graph. In this case, the *de Bruijn Graph* contains as many nodes as there are distinct kmers in the read dataset.

Sequencing errors can generate numerous novel distinct kmers that are present in only one or very few reads. This increases drastically the number of nodes in the graph. To avoid this, only kmers that are sufficiently covered in the dataset are represented in the graph, that is kmers having more than *T*_*sol*_ (solidity threshold) occurrences in the read dataset, hereafter called *solid* kmers.

The number of nodes, the number of edge per node, and the graph topology, have a strong impact on the size of the data structure. A given node is said to be branching if it has more than one in-going edges or more than one out-going edges. A simple path is then a path of nodes without any branching node. In order to efficiently store most of the reads, the graph should contain long simple paths such that the majority of reads will follow a simple path (without needing to store any bifurcation or difference). This is governed by two parameters, *k* and *T*_*sol*_.

Although both parameters are tunable by the user, the default mode of LEON does not require any user choice. The default *k* value is 31 and the optimal *T*_*sol*_ value is inferred automatically from the analysis of the kmer counts profile, with a method similar to the one used in KMERGENIE [17], and also briefly discussed in the Additional file 1: Section 2.

### Probabilistic *de Bruijn Graph*

A traditional implementation of a *de Bruijn Graph* requires a lot of memory. For example, hash table implementation similar to the one by Iqbal et al. [18],that stores for each node, a kmer and a byte containing the edges, requires at least $ 8 \lceil \frac {k}{32}\rceil + 1 $ bytes per node. This means approximately 27 GB for a human sized genome, which is largely prohibitive for compression purposes. Therefore a more lightweight implementation is required.

The notion of probabilistic *de Bruijn Graph* was first introduced by T.Brown et al. [19], and refers to a *de Bruijn Graph* represented as a Bloom filter. It was shown that the graph nodes can be encoded with as little as 4 bits per node, with the drawback of introducing false nodes and false branchings. Chikhi and Rizk [20] then also used a bloom filter to store the *de Bruijn Graph*. An additional structure storing critical false positives, rendered the *de Bruijn Graph* representation exact at a total cost of approximately 13 bits per node, then improved to 8 bits per node with cascading bloom filters [21].

The **Bloom filter** [16] is a space efficient hash-based data structure, designed to test whether an element is in a set. It is made up of a bit array initialized with zeros, and a set of hash functions. When inserting or querying for an element, its hash values are computed yielding a set of array positions. The insert operation corresponds to setting to 1 all these positions, whereas membership operation returns *yes* if and only if all the bits at these positions are set to 1. A *no* answer means the element is definitely not in the set. A *yes* answer indicates that the element may or may not be in the set. Hence, the Bloom filter has one-sided errors. The probability of false positives increases with the number of elements inserted in the Bloom filter.

Inserting the graph nodes in the bloom filter is sufficient to represent the *de Bruijn Graph*. Graph edges can be inferred by querying for the existence of all 4 possible successors of a given node.

For LEON’s compression purposes the main issue is the total graph size, while the exact representation of the graph is not a major issue: it only implies that additional bifurcation events may need to be stored for some reads. Therefore, a probabilistic *de Bruijn Graph* is chosen, since it provides both memory-efficient representation and reasonably fast construction of the graph: the list of solid kmers are simply inserted into a bloom filter.

There is a trade-off between the size of the bloom filter and its impact on the storage size of each read: a small bloom filter will take less space in the compressed file but will induce more storage space for each read. Since the bloom filter size is amortized across all reads, the optimal bloom filter size depends on the depth of sequencing (see Additional file 1: Section 5).

### Encoding the read sequences

The reference stored in the *de Bruijn Graph* does not contain all the necessary information to retrieve a given read. The idea is to store the minimum information required to reconstruct a read from the graph when decompressing. The data needed is an *anchor* kmer to indicate where to begin the reconstruction in the graph, a list of *bifurcations* to tell which path to follow, and the read size to know when to stop read reconstruction.

#### Dictionary of anchors

An *anchor* kmer is required to reconstruct a read from the graph. It is equivalent of read position in a reference genome for reference-based compression methods.

This is an important issue. A naive solution storing the raw kmer for each read would require to store, for example, *k* out of the total *l* nucleotides of a read, representing *k*/*l*=30/100 in a typical situation. This would severely limit the overall compression ratio.

LEON tackles this problem by reusing several times the same anchor kmer for different reads. Common anchor kmers are stored in a dictionary of kmers saved in the compressed file. Thus, an index in this dictionary is sufficient to encode a kmer anchor, requiring much less space than a kmer.

The selection procedure for the anchor kmer is as follows: each kmer of a read is considered as a putative anchor and queried in the dictionary of anchors. When one is found, the procedure stops and the anchor kmer is encoded as its index in the dictionary. If none is found, one *suitable* anchor kmer is selected in the read, then inserted in the dictionary. A *suitable* kmer is a solid kmer, i.e. a kmer that is also guaranteed by design to be a graph node. When no *suitable* kmers are found, the read cannot be mapped to the graph, it is encoded as a read without anchor.

#### Bifurcation list

The bifurcation list tells how the read is mapped to the graph, i.e. which path it follows whenever a bifurcation occurs. Since the anchoring kmer can be in the middle of the read, two bifurcation lists are needed, along with the sizes of the two paths. In practice, only read length and anchor position are encoded, from which the two paths sizes can be inferred. In the following, only the path at the right of the anchor is described, the other being symmetrical.

**Fig. 2:**
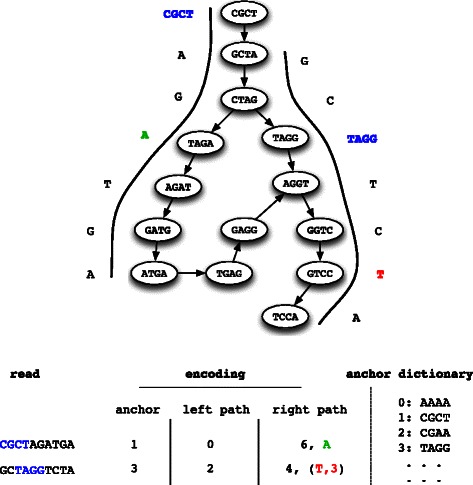
Schematic description of LEON’s path encoding. In the upper part, the mapping of two reads to the *de Bruijn Graph* is represented. Kmer anchors are shown in blue, bifurcations to (read on the left side) or difference from the graph (read on the right side) are respectively highlighted in green and red. In the bottom part, the corresponding path encodings for these two reads are shown: the index of the kmer anchor, and for each side the path length and bifurcation list



Starting from the anchor, the four possible kmer successors are queried in the *de Bruijn Graph*, and compared to the following kmer in the read. If only one successor exists and is the same as the kmer in the read, this is a simple path, nothing needs to be encoded. On the contrary, whenever an ambiguity occurs, such as several neighbors in the graph, the real nucleotide is added to the bifurcation list. It should be noted that, in general, the bifurcation position in the read is not required, since it is contained in the graph. However, in the special case of a simple path that is different from the read, both nucleotide and read position needs to be added. This is the case for instance for a sequencing error in the read. In this case, when decompressing, the error position cannot be inferred from the graph. The detailed construction mechanism is explained in Algorithm 1, and an encoding example is shown in Fig. 2.

#### Reads without anchor

**Fig. 3:**
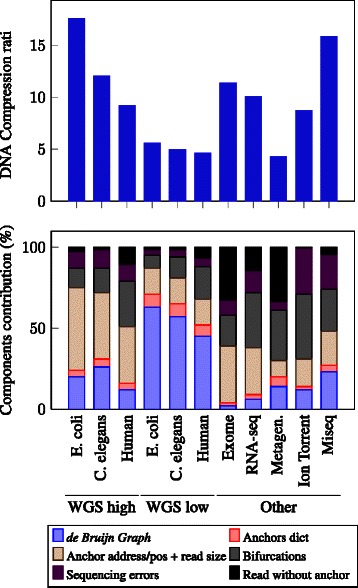
Components contribution in sequence compression. Sequence compression ratio (top) and relative contribution of each component in the compressed sequence stream (bottom) for diverse datasets. WGS high means high coverage (116, 70 and 102 x respectively), WGS low means down-sampling to 10x

Reads that cannot be mapped to the graph are simply encoded in the file with their raw sequence of nucleotides. This only happens if no kmer of the read is solid, i.e. if there is at least one sequencing error every *k* nucleotides or if the read is from a low covered region. Therefore, this is a rare event, it does not impact significantly the compression ratio (verified experimentally, see Fig. 3).

#### Arithmetic coding

All elements inserted in the compressed file (except for the bloom filter) are encoded with order 0 arithmetic coding [22]. LEON uses an individual model for each component (read size, anchor position, bifurcation list, raw nucleotides for un-anchored reads, dictionary of anchors), registering symbol frequencies and assigning fewer bits to frequent symbols.

#### Decompression

The main difference between the decompression and the compression processes is the reference building step that is not required during decompression. The decompression process starts by loading in memory the *de Bruijn Graph* and the anchor dictionary. For each read, the anchor kmer is obtained by a simple access to the dictionary of anchors. The anchor position and the read size are then decoded to know how many nodes of the *de Bruijn Graph* need to be explored in each direction (left and right paths). The process to recover the read sequence in each direction starting from its anchor is similar to the one described in algorithm 1. We first check in the bifurcation list if we are at a position where the nucleotide is different from any path in the *de Bruijn Graph* (typically the case of a sequencing error). In this case, we add to the read the next nucleotide of the bifurcation list. In other cases, the successive nucleotides are obtained from the walk in the *de Bruijn Graph* and whenever a bifurcation is encountered, the path to choose is given by decoding the next nucleotide of the bifurcation list.

### Quality compression

It has been observed that *lossless* quality compression methods are reaching a *wall*, *i.e.* a maximum compression rate that cannot be exceeded [5]. This comes from the nature of the quality stream, meaning that it is too noisy to be efficiently compressed on a lossless basis. Moreover, the usefullness of such a large panel of quality scores is not self-evident. Most downstream NGS analysis will ignore the fact that a nucleotide has a probability of error of 5.0∗10^−4^ rather than 3.9∗10^−4^.

For these reasons, a *lossy* compression scheme was chosen for LEON. Similar to work by Janin et al. and Yu et al. [14, 15], we use the evidence contained in the reads to smooth the quality scores. For this, we capitalize on the information already computed during the DNA sequences compression step, *i.e.* the set of *solid* kmers stored in the Bloom filter. Based on the assumption that nucleotides being covered by a sufficient number (*σ*) of solid kmers can safely be considered as error-free, they are being assigned an arbitrarily high quality value (‘@’). However, upgrading low quality scores to higher values is more risky for downstream NGS analysis than replacing already high scores, as this may incur, for instance, false positives SNP-calls. To alleviate this risk, we require a higher number *σ* of solid kmers in order to trigger quality replacement for these low quality values.

In details, the procedure is as follows. (1) We first truncate all quality scores above a given threshold (qualities higher than ‘@’ are replaced by ‘@’). (2) All positions that are covered by at least *σ* solid kmers have their quality score replaced by ‘@’. (3) The quality stream is compressed with the zlib library. The *σ* parameter is computed as follows: with *δ*=*`* *@* ^′^−original quality, we set *σ*=2 if *δ*≤10, and *σ*=*δ*−5 otherwise.

This approach is obviously *lossy* since it changes the original qualities. However, modifying the quality values based on the information extracted from the reads, means that some quality scores are actually *corrected*. This can be viewed as an amelioration instead of a loss, and should explain the improvements of downstrean NGS analysis already discussed in RQS results [15]. In this context, we explore the effect of our quality smoothing procedure on SNP-calling in Table 2.

### Implementation

#### GATB library

The GATB library (http://gatb.inria.fr/) was used to implement LEON [23]. This library provides an API for building and navigating a *de Bruijn Graph* and its implementation, based on the Bloom filter and the constant-memory kmer counting algorithm introduced by Rizk and Chikhi [24], and later improved by new methods introduced by Deorowicz et al. [25], i.e. minimizer-based kmer partitioning and (*k*,*x*)-mers counting.LEON is able to compress Fasta or Fastq files, and has the option to compress quality scores in lossless or lossy mode.

#### Header compression

To compress the sequence headers, a classic compression approach was used. A typical header string can be viewed as several fields of information separated by special characters (any character which is neither a digitn nor alphabetic). Most of these fields are identical for all reads (for instance, the dataset name or the size of the reads). The idea is to store fixed fields only once and efficiently encode variable fields. A short representation of a header can be obtained using its previous header as reference. Each field of the header and its reference are compared one by one. Nothing needs to be kept when fields match. When differences occur, either the numerical difference or the size of the longest common prefix are used to shorten the representation. The resulting short representation is encoded using an order 0 arithmetic coding.

#### Complexity

If we omit the kmer counting step, LEON performs compression and decompression in one single pass over the reads. For a given read, selecting the anchor and building the bifurcation list requires a number of operations that is proportional to the number of kmers in the read. Both compression and decompression processes have running times proportional to the read count multiplied by the average number of kmers per read, that is a time complexity linear with the size of the dataset.

It is important to note that decompression is faster than compression. The time consuming kmer counting step is not performed during decompression since the *de Bruijn Graph* is stored in the compressed file.

Two main structures are maintained in main memory during compression and decompression. The bloom filter can use up to *G*∗*b* bits for storing solid kmers where *G* is the size of the target genome and *b* is the number of bits per solid kmers (typically *b* is set to 12). During anchor selection, the minimum requirement is to choose a solid kmer as anchor. It means that like the bloom filter, the maximum number of anchors that can be inserted in the dictionary is *G*, the size of the genome. The important thing to notice is that the amount of memory needed by LEON is not related to the size of the input file but proportional to the size of the target genome.

#### Parallelization

To allow our method to fully benefit from multi-threading, reads of the input file are split in blocks of *n* reads. Each block is then processed independently of the others by a given thread.

Parallelization speed-up is shown in Additional file 1: Figure S3.

### Datasets and tools

#### NGS datasets

LEON’s performance was evaluated on several publicly available read datasets. Main tests and comparisons were performed on whole genome sequencing (WGS) Illumina datasets with high coverage (more than 70x), from three organisms showing a large range of genome sizes and complexities: a bacteria *E. coli* (G=5 Mbp), a nematode *C. elegans* (G=100 Mbp) and a human individual (G=3 Gbp). The largest file tested is the WGS human one with 102x coverage resulting in an uncompressed fastq file size of 733 GB. To evaluate the impact of sequencing depth, these datasets were then randomly down-sampled. Additionally, other types of sequencing protocols and technologies were tested, such as RNA-seq, metagenomics, exome sequencing or Ion Torrent technology. Detailed features and accession numbers of each dataset are given in Additional file 1: Table ST1.

#### Other tools and evaluation criteria

Several compression software were run on these datasets to compare with LEON, from best state-of-the-art tools to the general purpose compressor GZIP (Additional file 1: Table ST2). Tools that are able to compress the whole fastq files and that allow to properly handle paired reads were preferentially chosen. SCALCE and MINCE that re-order reads were run with their option to keep paired reads together. Additionally, being the best read-reordering tool, Orcom was kept for reference. However, it cannot be directly compared to other tools as it loses the read pairing information. Manually concatenating paired reads before compression is possible for ORCOM, however this leads to poor compression ratio. Hence we decided to show ORCOM results only in the mode that lose paired read information.

LEON and concurrent tools were compared on the following main criteria: (i) compression ratio, expressed as the original file size divided by the compressed file size, (ii) compression time, (iii) de-compression time and (iv) main memory used during compression.Since the main compared feature is compression ratio, concurrent tools were tuned for maximum compression when possible. LEON was always used with default parameters. Moreover, since LEON’s default mode for quality score compression is lossy, other tools were also run in a lossy configuration for quality scores (see Additional file 1: Section 4.1 for additional details and used command lines).

All tools were run on a machine equipped with a 2.50 GHz Intel E5-2640 CPU with 12 cores, 192 GB of memory.All tools were set to use 8 threads.

#### Lossy quality compression evaluation

Lossy quality compression ratios cannot be compared without also taking into account the impact of the lossy transformation of the quality stream on downstream analysis, for instance SNP-calling accuracy. An experiment on a read dataset from the “1000 genomes project” (phase 1 release) was performed. More specifically, SNPs were called before and after several lossy quality transformations on a low coverage Illumina read sequences dataset, corresponding to the human chromosome 20 (HG00096 individual, SRR062634).

Five reference-free, lossy quality compression tools were tested together with LEON, each of them being representative for a particular category of methods: FASTQZ that lowers the number of bins of the quality spectrum, LIBCSAM and FQZCOMP that smooth qualities within a block, and RQS as a tool that, similarly to LEON, uses information extracted from the DNA sequences. The SNP-calling results obtained for the original qualities were compared with those for the transformed qualities, and with those obtained for a naive quality transformation where all qualities are replaced by an arbitrarily chosen high score value, ’H’ (this corresponds to the extreme case where all qualities are discarded). Reads were mapped with BWA, then samtools mpileup followed by bcftools procedure was used to call SNPs and to generate the VCF files [26, 27]. To assess the number of SNPs that were lost in each quality transformation process, as well as the ones that were potentially found in addition to those detected with the original qualities, each VCF file was compared to what we consider to be the reference SNP set, *i.e.* the VCF file that was produced by the “1000 genomes project” on the same sequencing data. The precision and recall measures were computed with respect to the VCF reference file, with the same procedure detailed by the authors of LIBCSAM [13]. For RQS that only transforms qualities, and for LEON (that compresses the header and the sequence but only transforms the quality part), the quality streams were compressed with the general purpose compression tool GZIP.

## Results

### Impact of the parameters and *de Bruijn Graph* false positives

The compression ratio of LEON crucially depends on the quality of the reference that is built *de novo* from the reads, the probabilistic *de Bruijn Graph*.

In order to evaluate the impact of using an approximate *de Bruijn Graph* compared to an exact representation, the compression ratio of LEON was computed for several sizes of bloom filters expressed as a number of bits per node. The larger the bloom filter, the fewer the false positives but the more space is needed to store it. Figure S4 in Additional file 1 shows that the optimal trade-off lies around 10 bits per solid kmer, for the 70x *C. elegans* dataset. It also demonstrates that correctness of *de Bruijn Graph* is not essential for compression purposes.

The kmer size and the minimal abundance threshold (parameters *k* and *T*_*sol*_ respectively) also impact the compression ratio, as they control the number of nodes and the topology of the exact *de Bruijn Graph*. In fact LEON compression ratio proves to be robust to variations of these parameters around the optimal values (see the results on varying these parameters in Additional file 1: Figures S1 and S2). Therefore, LEON can safely be used with its default parameters.

### DNA compression ratio with respect to dataset features

**Fig. 4:**
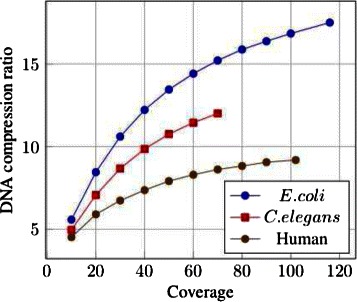
Sequence compression ratios by coverage. Compression ratios obtained by LEON on the sequence stream, with respect to the sequencing coverage of the datasets. The three WGS datasets were down-sampled to obtain lower coverage

Figure 4 shows that the compression ratio increases with the sequencing depth. Obviously, the more redundant information is contained in the file, the more LEON can compress it. This is due to the fact that the space occupied by the Bloom filter does not depend on the sequencing depth and is rapidly negligible compared to the initial space occupied by the reads when coverage increases (see also Fig. 3). Notably, the compression factor depends also on the sequenced genome size and complexity, with better compression for the small and less complex bacterial genome. In this case the *de Bruijn Graph* contains more simple paths and bifurcation lists are smaller.

Figure 3 shows the relative contributions of each component of the DNA compressed stream for diverse datasets. For WGS datasets, this confirms that the relative contribution of the Bloom filter is low for high coverage datasets, but prohibitive for low coverage datasets (10x).

For other types of datasets, the relative contributions vary greatly. For instance, the exome dataset is well compressed since the coverage is very high (more than 1000x) on a very small reference (exons representing around 1 % of the human genome). However, as the capture is noisy and some reads fall outside exons, an important part of the compressed file is taken by un-anchored reads.

For the RNA-seq and metagenomic datasets, the bifurcation and un-anchored reads components represent the major part of the compressed DNA stream sizes. This is due to the heterogeneous sequence abundances in these kinds of datasets. In such cases, sequencing errors cannot be identified solely based on the kmer abundances and the solidity threshold is less effective in simplifying the graph. For instance in the case of RNA-seq, highly transcribed genes are likely to generate parts of the *de Bruijn Graph* with a high density of branchings, the majority of them corresponding to sequencing errors. Conversely, in the metagenomic dataset, numerous species have a low abundance in the sample and their genome is not represented in the *de Bruijn Graph*, resulting in a high number of un-anchored reads.

Among the tested datasets, three correspond to the same target species (*E. coli*), sequenced with similar sequencing depths (∼115x) but with different sequencing technologies or protocols : Illumina HiSEq 2000, Illumina MiSeq and Ion-Torrent. In Fig. 3, one can observe that this factor impacts the DNA compression ratio and the relative contributions of each component. The Ion-Torrent dataset has the lowest compression ratio and this is mainly due to the bifurcation and sequencing errors components. This is explained by the sequencing errors that are mostly insertions and deletions, which are not well handled by the current bifurcation algorithm (an insertion or deletion implies the rest of the read will be encoded as errors), contrary to substitution errors. In the Illumina MiSeq protocol, reads are longer than in the classical HiSeq (250 vs 100 pb). Consequently, for the same amount of DNA, there are fewer reads and therefore fewer anchors to be encoded. This explains the great difference in the relative contribution of the anchor address component. Note that overall DNA compression ratio are roughly similar between both protocols, but this is due to a higher number of sequencing errors in this particular MiSeq dataset. Since the technologies are evolving to produce longer reads with fewer sequencing errors, this suggests that LEON compression ratio will easily fit the technology evolutions.

Lastly, because of the anchor selection procedure, initial read order may theoretically impact compression ratio. However, test showed that LEON compression ratio only varies slightly when changing read order, generally below 1 % variation.

### Comparison with other tools

For high coverage WGS datasets, LEON obtains the best compression ratio for the whole FASTQ file, i.e. sequence, header and quality streams combined, in comparison to other compression software (see Table 1). In particular, with respect to the most used tool, GZIP, LEON compressed file can be up to 7 times smaller than the GZIP one for high coverage datasets. In the large human dataset case, we can save up to 686 GB (the file size drops from 733 GB to 47 GB).

**Table 1:** Compression features obtained for the three high coverage WGS datasets with several compression tools. Total compression ratio is the compression ratio (original size / compressed size) of the whole FASTQ file, header, sequence and quality combined

Method	Compression ratio				Compression		Decompression	
	Total	Header	Base	Quality	Time (s)	Mem. (MB)	Time (s)	Mem. (MB)
SRR959239 - WGS *E. coli* - 1.4 GB - 116x								
gzip	3.9	—	—	—	179	1	13	1
dsrc*-lossy*	7.6	—	—	—	9	1942	13	1998
fqzcomp*-lossy*	17.9	35.2	12.0	19.6	73	4171	74	4160
fastqz*-lossy*	13.4	40.8	14.1	8.7	255	1375	298	1375
leon*-lossy*	**30.9**	45.1	17.5	59.3	39	353	33	205
scalce*-lossy*	9.8	21.4	8.3	9.2	62	2012	35	2012
quip	8.4	29.8	8.5	5.3	244	1008	232	823
mince	—	—	16.7	—	77^*#*^	1812	19^*#*^	242
orcom*	—	—	34.3*	—	10^*#*^	2243	15^*#*^	197
SRR065390 - WGS *C. elegans - 17 GB - 70x*								
gzip	3.8	—	—	—	2145	1	165	
dsrc*-lossy*	7.9	—	—	—	67	5039	85	5749
fqzcomp*-lossy*	12.8	54.2	7.6	15.0	952	4169	1048	4159
fastqz*-lossy*	10.3	61.9	7.3	8.7	2749	1527	3326	1527
leon*-lossy*	**21.3**	48.6	12.0	32.9	627	1832	471	419
scalce*-lossy*	8.2	34.1	6.5	7.2	751.4	5309	182.3	1104
quip	6.5	54.3	4.8	5.2	928	775	968	771
mince	—	—	10.3	—	1907^*#*^	21825	387^*#*^	242
orcom*	—	—	24.2*	—	113^*#*^	9408	184^*#*^	1818
SRR345593/SRR345594 - WGS human - 733 GB - 102x								
gzip	3.3	—	—	—	104,457	1	9124	1
dsrc*-lossy*	7.4	—	—	—	2797	5207	3598	5914
fqzcomp*-lossy*	9.3	23.2	5.3	15.0	39,613	4169	48,889	4158
fastqz(a)	—	—	—	—	—	—	—	—
leon*-lossy*	**15.6**	27.5	9.2	26.8	40,766	9556	21262	5869
scalce(b)	—	—	—	—	—	—	—	—
quip	6.5	54.3	4.8	5.2	52,854	776	46594	775
mince(a)	—	—	—	—	—	—	—	—
orcom*	—	—	19.2*	—	29,364^*#*^	27505	10,889^*#*^	60,555

The following columns indicate the ratio for each individual component, when available. Running time (in s) and peak memory (in MB) are given for compression and decompression. All tools were used without a reference genome. Best overall results are in bold

^a^Program does not support variable length sequences

^b^SCALCE was not able to finish on the large WGS human dataset

*-lossy* suffix means the method was run in *lossy* mode for quality scores compression

^*^Stars indicate that the given program changes read order and loses read-pairing information, and thus cannot be directly compared to other tools

Running time with ^#^ is on DNA sequence only

Best overall results are in bold

Interestingly, although QUIP is similar in approach to LEON, results in terms of sequence compression ratio are much lower than LEON. This can probably be explained by the large amount of reads that could not be mapped to the assembled contigs, either because they were incomplete or too fragmented. As expected, ORCOM, which allows read-reordering, achieves the highest sequence compression ratios. However, it looses important read pairing information and thus cannot be directly compared to *lossless* methods. Moreover, it only compresses the DNA sequence part and completely discards header and quality scores. MINCE and SCALCE that both re-order reads but keep read pairing information have a lower compression ratio than LEON on the DNA sequence. It seems than keeping read pairing information without degrading compression ratio is not a simple task for read-reordering methods.

To be on par with LEON lossy quality scores compression, other tools were also run in a lossy compression mode when available (see command lines in Additional file 1: Table ST2). LEON achieves much higher compression of quality scores than other tools, 26.8 on the human dataset, compared to 15x for FASTQZCOMP.

**Fig. 5:**
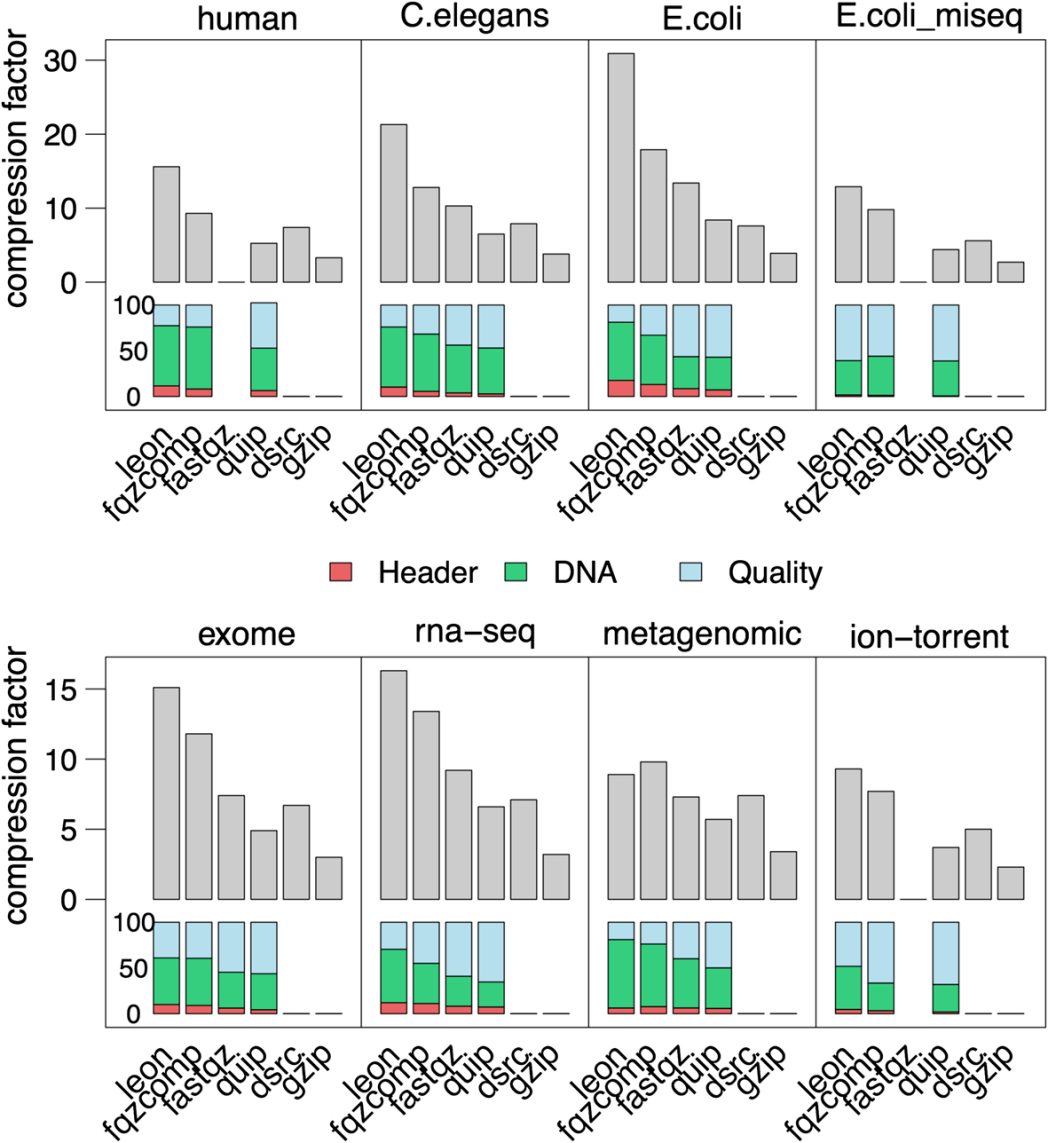
Compression ratios comparison. Comparison of compression ratios between *de novo* compression software for diverse datasets. On top, overall compression factor (orignal file size / compressed file size). The bottom part represents space distribution between header, sequence and quality scores (respectively in red, green and blue)

Additional comparisons on other types of datasets are shown in Fig. 5. LEON is better than other tools on all datasets, except on the metagenomic one where all tools perform roughly equally bad. In general, the sequence stream takes the most space in the whole compressed file for all tools. Interestingly, this is not the case for the Miseq and ion-torrent datasets. They suffer from higher sequencing error rates, which impact more the quality smoothing than the sequence compression.

Concerning running times, DSRC is by far the fastest method. It achieves compression ratios generally lower than other methods, but still up to two times better than GZIP. It is a good choice when running time is the major concern. Apart from DSRC, LEON compression time is about the same order of magnitude as other methods compressing the whole FASTQ file, and a bit faster for decompression.

Regarding memory, contrary to other tools that use fixed memory resources, the memory used by LEON depends on the genome size, with less than 2 GB for a medium genome such as *C. elegans*. Importantly, it remains reasonable for a human genome with 9.5 GB, making LEON still usable on desktop computers.

### Impact of lossy compression of qualities

**Fig. 6:**
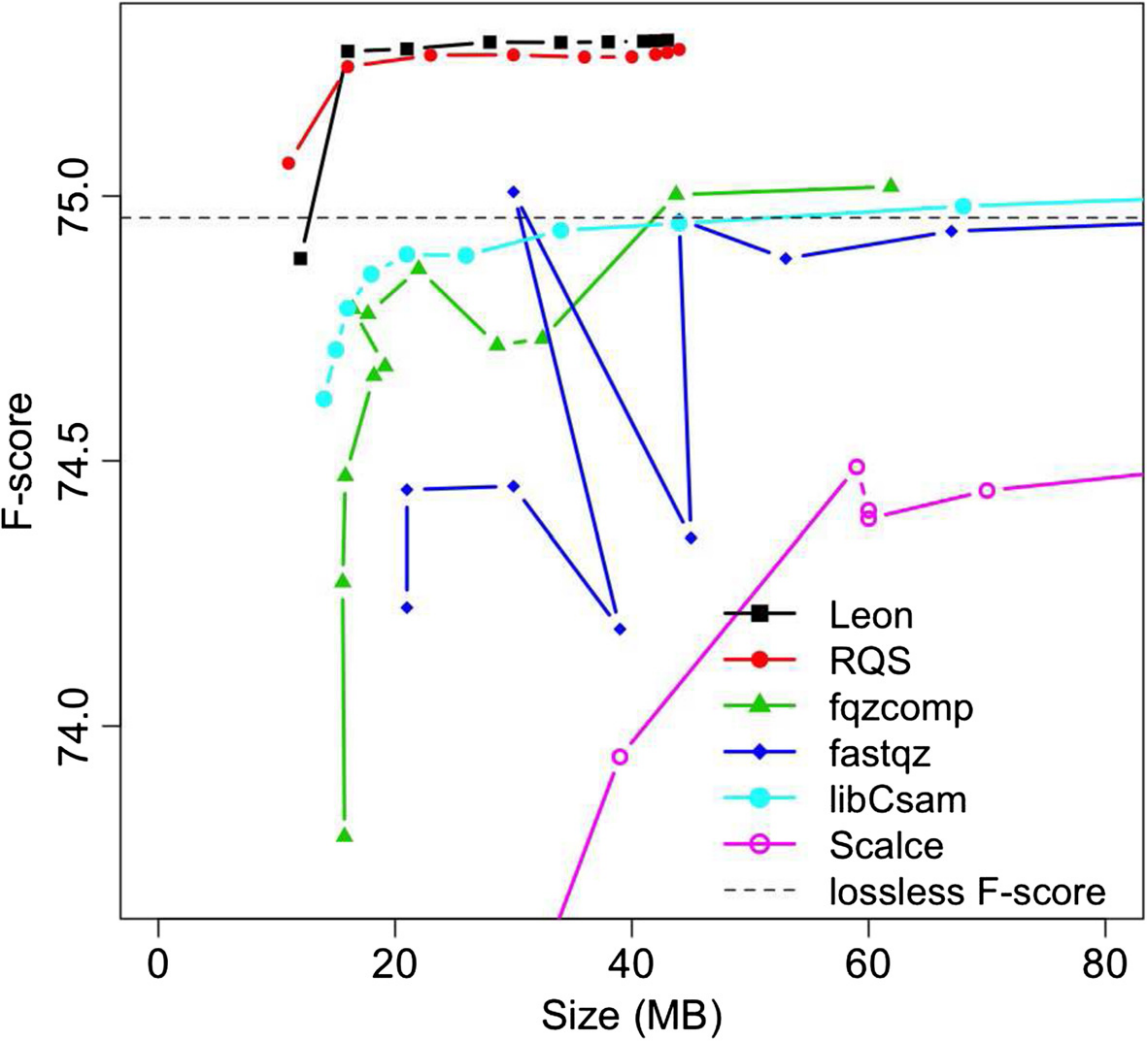
Compression / accuracy trade-off for quality compression. Impact of *lossy* compression methods of quality scores on SNP calling, for a human chromosome 20 (HG00096 individual, SRR062634) compared to a gold standard. Each line represents the F-score/compressed size trade-off for a method, the higher the line, the better. The dashed line represents the F-score obtained by the original fastq file and by lossless compression methods

The impacts of several lossy quality compression schemes were evaluated by measuring the SNP-calling accuracy. Results are summarized in Table 2 and Fig. 6.

**Table 2:** SNP calling precision/recall test on data from human chromosome 20, compared to a gold standard coming from the “1000 genomes project”

HG00096 chrom 20			
Prog	Precision	Recall	Compression ratio
*lossless*	85.02	67.02	2.95
SCALCE	85.15	66.13	4.1
FASTQZ	85.46	66.63	5.4
LIBCSAM	84.85	67.09	8.4
FQZCOMP	85.09	66.61	8.9
LEON	**85.63**	**67.17**	11.4
RQS	85.59	67.15	12.4
*no quality*	57.73	68.66	-

*No quality* means all qualities were discarded and replaced by ’H’. The ratio is given by the original quality size divided by the compressed size. For the *lossless* line, the best compression ratio obtained by lossless compression tools is given (obtained here with FQZCOMP). Results are ordered by increasing compression ratio

Best overall results are in bold

In Table 2 we compared the compression ratios obtained by the five lossy quality compression tools with parameters producing precision and recall values close to those obtained for the reference lossless case. The *lossless* and the *no quality* results give precision/recall scores of extreme cases where, respectively, the original quality scores are kept or completely discarded. Moreover, the original qualities file compressed with FQZCOMP tool (the lossless compression tool that gives the best compression ratio on this data) gives a lower bound in terms of compression ratio. The parameters used for these tests, given in Table ST2 of Additional file 1, were chosen to yield a good compromise between precision/recall and compressed size.

The results in Table 2 show that, unsurprisingly, naive smoothing (*no quality*) leads to high recall but very poor precision. Moreover, FASTQZ, FQZCOMP and LIBCSAM have both lower precision/recall scores and compression ratios than LEON and RQS.

This confirms our initial hypothesis that smoothing qualities based on the information extracted from the reads is more effective than reducing the quality spectrum with generic transformations. Moreover, the precision/recall results of RQS and LEON corroborate the observation made in [15, 28] regarding the ability of such tools to locally correct the data and thus to enhance the SNP-calling process.

Whereas in Table 2 we choose, for each tool, one set of parameters giving a good compromise between precision/recall and compressed size, in Fig. 6 we analyze a wide range of parameters affecting the trade-off between compression ratios and SNP-calling results (measured with the F-score). Figure 6 shows the F-score as a function of the compressed size.

For LEON and RQS the kmer solidity threshold (*T*_*sol*_) was varied. For FQZCOMP, FASTQZ and LIBCSAM the parameter governing the amount of quality score modified was varied. As expected, for LEON and RQS the F-score increases when the compressed size increases, and is above that of the other tools and also above that of the original file (indicated with a dashed line). Even though coherent, LIBCSAM F-score results are clearly below those of LEON and RQS. On the other hand, FQZCOMP and especially FASTQZ exhibit strange behavior, as parameters that should yield smaller compressed files and lower F-scores sometimes achieve bigger compressed sizes and lower F-scores.

## Discussion

In this article, we introduced a new method for reference-free NGS data compression. Whereas the QUIP approach is building a *de novo* reference with traditional assembly methods, we use a *de Bruijn Graph* as a *de novo* reference. This allows skipping the computationally intensive and tricky assembly step, and also allows to map more reads on the graph than would be possible on a set of *de novo* built contigs. Our approach also yields better compression ratios than context model based methods such as FASTQZ or FQZCOMP, which, in a way, also learn the underlying genome from the context. This can be explained by the larger word size used by LEON. Thanks to the probabilistic *de Bruijn Graph*, our method is able to work with large kmers, whereas context models are limited to order-14 models due to memory constraints.

The development of an API in the GATB library to read the LEON format on-the-fly without full decompression on disk is under development and will facilitate usage by other tools based on GATB (that could use it as a native input format). Moreover, the LEON compressed file contains more information than just the raw list of reads: the included *de Bruijn Graph* can be directly re-used by other software. For example, the TAKEABREAK and DISCOSNP software [29, 30] detecting polymorphisms from the *de Bruijn Graph* will be able to take as input a LEON file and save significant time from the graph construction step. In this way, LEON can be seen as more than just a compression tool, as it also pre-processes data for further NGS analysis.

Further developments to enhance LEON performance and functionalities are also considered. First, if reordering reads is acceptable for the user, grouping reads with the same anchor would allow to store the anchor once for many reads and save significant space. However, read-reordering strategy is acceptable in our opinion only if read pairing information is preserved, which is not the case of current read-reordering methods. With LEON method, since paired reads are close in the graph, it may be possible to encode paired reads together, by also encoding the path in-between reads. This would allow read reordering without losing read pairing information. Secondly, the detection of insertion and deletion errors could boost substantially the compression ratio of datasets issued from novel sequencing technologies (Ion Torrent or Pacific Bioscience). Moreover, our approach makes it possible to deal with multiple datasets efficiently. It would be straightforward to store the *de Bruijn Graph* only once for several datasets sequenced from the same organism for instance, and thus improving compression ratio.

Lastly, our approach bears some similarities with error correction methods. When reads are anchored to the graph, some sequencing errors are clearly identified and saved in the file for the decompression. It could be combined with more powerful error detection algorithms to provide state-of-the art error correction, for example with the BLOOCOO^1^ tool already implemented with the GATB library [23]. It would then be straightforward to propose an option when decompressing the file, to choose between lossless sequence decompression mode, or with the sequencing errors corrected.

## Conclusions

We introduced LEON, an all-in-one software for FASTQ file compression that handles DNA, header and quality scores. LEON uses the same data structure for both DNA and quality scores compression, a *de Bruijn Graph* compactly stored in a Bloom filter. The quality compression scheme is lossy, allowing for good overall compression ratios, without losing the essential quality information and thus not hampering downstream NGS analysis. LEON achieves higher compression ratios than other state-of the art tools and seems robust regarding diverse types of data.

## Endnote

^1^ http://gatb.inria.fr/software/bloocoo/

## Additional file

Additional file 1 **Supplementary material.** This supplementary file contains full details of datasets used and command line parameters of tools benchmarked, as well as additional test results concerning the impact of parameters *k* and *T*_*sol*_ on LEON. (PDF 116 kb)

## Abbreviations

NGS: next-generation sequencing
SNP: single-nucleotide polymorphism
BWT: Burrows-Wheeler transform
LCP: Longest common prefix
WGS: Whole-genome sequencing
