# Supplementary material for: Reference-free compression of high throughput sequencing data with a probabilistic de Bruijn graph
Source: BMC Bioinformatics. 2015 Sep 14;16:288. doi: 10.1186/s12859-015-0709-7 (PMC4570262; doi:10.1186/s12859-015-0709-7)
Supplement: Supplementary file 1 — Supplementary material. This supplementary file contains full details of datasets used and command line parameters of tools benchmarked, as well as additional test results concerning the impact of parameters k and T sol on LEON. (PDF 116 kb) [file 12859_2015_709_MOESM1_ESM.pdf]

Supplementary Material for

Compression of high throughput sequencing data with a probabilistic de Bruijn graph

Gaëtan Benoit [1], Claire Lemaitre [1], Dominique Lavenier [1], Erwan Drezen [1], Thibault Dayris [2], Raluca Uricaru [2,3], Guillaume Rizk [1,*]

[1] *INRIA/IRISA/GenScale, Campus de Beaulieu, 35042 Rennes cedex.*
[2] *University of Bordeaux, CNRS/LaBRI, F-33405 Talence, France.*
[3] *University of Bordeaux, CBiB, F-33000 Bordeaux, France.*
[*] *Corresponding author. Email Guillaume.Rizk@inria.fr*

Contents

1 Description of the sequence datasets **2**

2 Impact of parameters k and T_{sol} **2**

3 Parallelization speed-up **4**

4 Comparisons with other compression software **5**

4.1 Data formats and command line arguments 5

5 Theoretical estimation of the optimal bloom filter size **5**

1 Description of the sequence datasets

All read datasets used in the main paper are publicly available in the Sequence Read Archive (SRA) and were downloaded either from the NCBI or EBI web servers. Description of each dataset along with its SRA accession number is given in Table ST1.

SRA Accession	Org.	Type	Platform	R. size	Read count	Base count	Cov.	Fastq size
SRR959239	*E. coli*	WGS	Illumina	98	5.4M	526.5 Mbp	116x	1.4 GB
SRR065390	*C. elegans*	WGS	Illumina	100	67.6M	6.2 Gbp	70x	22.6 GB
SRR345593/SRR345594	human	WGS	Illumina	101	3040M	304.0 Gbp	102x	733 GB
SRR359098/SRR359108	human	exome	Illumina	100	779M	78.0 Gbp	$\sim 1300x$	203 GB
SRR445718	human	RNA-seq	Illumina	100	32.9M	3.3 Gbp	–	11 GB
SRR857303	*E. coli*	WGS	Ion Torrent	195	2.6M	0.5 Gbp	109x	1.2 GB
SRR1519083	microorg.	metagenome	Illumina	100	59.7M	6 Gbp	–	16GB
SRR1870605	*E. coli*	WGS	Illumina MiSeq	242	2.2M	543.2 Mbp	119x	1.1GB

Table ST1: Read dataset description. WGS stands for Whole Genome Sequencing.

2 Impact of parameters k and T_{sol}

The kmer size and the minimal abundance threshold, ie. parameters k and T_{sol} respectively, impact Leon compression ratio, as they control the number of nodes and the topology of the *de Bruijn Graph*. Leon performance was then computed for varying values of these parameters for the *C. elegans* WGS dataset.

The T_{sol} parameter is inferred automatically from the histogram of kmer abundances (telling the number of kmers of each abundance). The following heuristic is used. The histogram is first smoothed, then we search for the position of the first increase, and the maximum value attained after that. We then look for the index of the minimum value between this first raise and this maximum. This index is the automatically inferred threshold, and roughly corresponds to the valley between erroneous kmers and “genomic” kmers. We also ensure that no more than 25 % of the distinct kmers are below the threshold, and that the threshold is $>= 3$.

Figure S1 shows that the compression ratio is robust to variations of the T_{sol} parameter around its optimal value. Importantly, the automatically inferred value, 8, gives a compression ratio very close to the optimal one. However, with more extreme values the compressed file size can drastically increase. For instance, if no filtering at all of low coverage kmers is performed, the compression ratio is divided by two (from 12.0 to 5.8), demonstrating the

importance of removing sequencing errors in the reference *de Bruijn Graph*. In this case, all reads can be mapped perfectly to the graph, but the size of the graph and of the bifurcation lists are important too. Conversely, if the threshold is too high, removing too many genomic kmers, the compression ratio drops since the reference is incomplete and many more reads cannot map to it. Regarding quality scores compression ratios, a higher T_{sol} value means fewer scores will be considered as good enough to be replaced by a high value '@', therefore quality compression ratios keeps decreasing with a higher T_{sol}. However, we observe a plateau near the optimal value for sequence compression, which probably corresponds to values distinguishing erroneous of solid kmers.

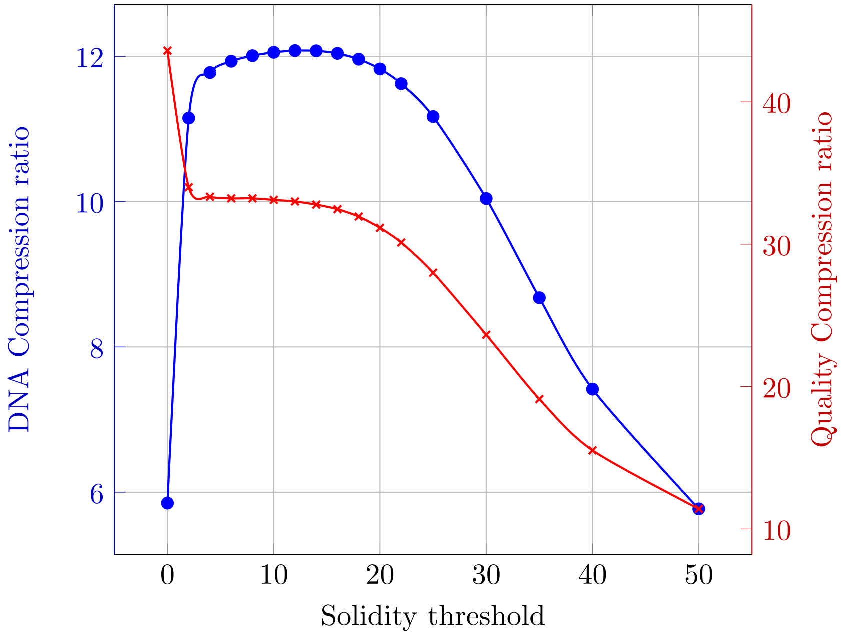

Figure S1: Variations of the compression ratio obtained by Leon on the *C. elegans* WGS dataset for varying values of the minimal abundance threshold (parameter T_{sol}). Leon was run with $k = 31$.

Similarly, Figure S2 shows that there is also an optimal value for the k parameter. For small k (typically $k < 20$ for the *C. elegans* dataset), many kmers are not unique in the genome, generating numerous branchings in the *de Bruijn Graph*. Even if the latter is much smaller in size with small k, this does not compensate for the numerous bifurcations to store for each read. Higher k value will also mean a larger dictionary of anchors and more unanchored reads. Consequently, this parameter depends on the complexity of the genome. For

large genomes with numerous repeats, larger k should be preferred, but a trade-off must be found to compensate compression ratio with running time, since the counting step time can increase with k.

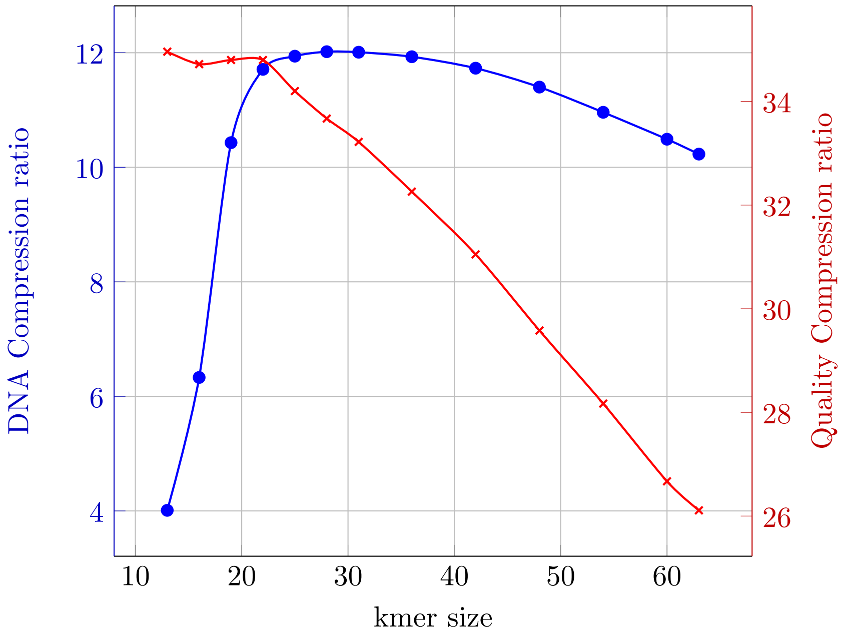

Figure S2: Variations of the compression ratio obtained by LEON on the *C. elegans* WGS dataset for varying values of kmer size (parameter k). The T_{sol} parameter was inferred automatically.

3 Parallelization speed-up

Figure S3 shows LEON execution time using 1 to 24 threads. The platform used is a 2.50 GHz Intel E5-2640 CPU with 12 cores (24 logical cores with hyper-threading). It should be noted that LEON seems to benefit highly from Intel hyper-threading. We suspect this is the case because LEON's major operations are queries inside a bloom filter, which induce many memory cache misses. Memory cache misses induce latency, usually well hidden by hyper-threading.

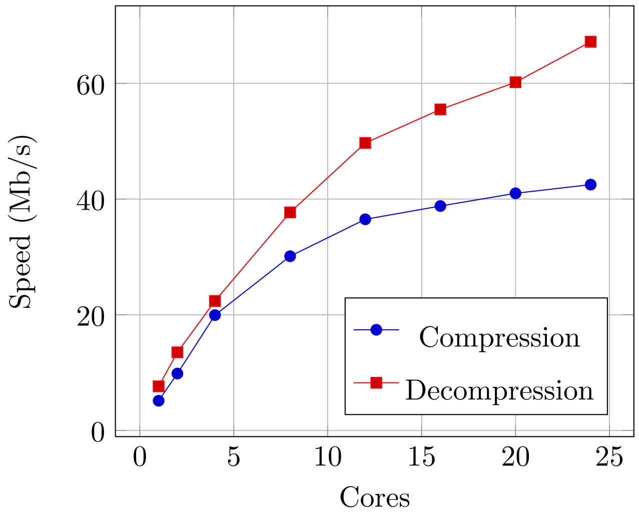

Figure S3: Speed of compression and decompression with respect to the number of used CPU threads, for the 70x WGS *C. elegans* dataset.

4 Comparisons with other compression software

4.1 Data formats and command line arguments

Software used to compare to LEON are described in Table ST2.

Since LEON default mode is lossy for quality scores, other tools were also run in a lossy mode when possible.

5 Theoretical estimation of the optimal bloom filter size

LEON uses a probabilistic *de Bruijn Graph*, i.e. all kmer nodes of the graph are inserted in a bloom filter. The false positive rate of the bloom filter will induce false branching in the graph, meaning extra bifurcation events that will need to be stored in the compressed file. Therefore, an optimal trade-off needs to be found: a large bloom filter will take more space in the file but will save space for read storage (and conversely).

The false positive rate $\mathcal{F}$ of a bloom filter can be approximated by $\mathcal{F} = f^r$ with $f = 0.5^{\log 2} \approx 0.618$ and $r = \frac{\text{bloom size}}{\text{nb elements inserted}}$ the number of bits per element inserted in the bloom filter [10].

With $\mathcal{G}$ the size of the target sequenced genome (i.e. approximately the number of nodes in the *de Bruijn Graph*) and $\mathcal{D}$ the average kmer abundance (somehow related to the depth of

Sotware	Ref.	Version	Mode	Command line
Leon	–	1.0.0	default	`leon -file file.fastq -c -nb-cores 8`
Fastqz	[1]	1.5	slow	`fastqz c3 file.fastq file.fastq.fastqz`
Fqzcomp	[1]	4.6	slow	`fqz_comp -n2 -Q3 -s8+ -b file.fastq file.fastq.fqzcomp`
Quip	[2]	1.1.8	ass.	`quip -i fastq -a file.fastq`
Orcom	[3]	1.0		`orcom_bin e -ifile.fastq -obin_tmp -t8`
				`orcom_pack e -ibin_tmp -oout_orcom -t8`
Dsrc	[4]	2.00	slow	`dsrc c -t8 -m2 -l file.fastq file.fastq.dsrc`
gzip	[5]	1.3.12		`gzip file.fastq`
Scalce	[6]	2.7		`scalce -c bz -o result -p 20 -T 8 -r file_1.fastq`
Mince	[7]	0.6.1		`mince -p 8 -e -l IU -1 file_1.fastq -2 file_2.fastq -o resu`
RQS	[8]	0.1.0		`generate_dict 5 dict.db file.fsam`
				`sparsify dict.db file.fsam`
				`threshold '@' file.fsam.filtered`
libCsam	[9]	08000b5		`CompressQual ./hg96.sam -q 1 -l 4`

Table ST2: Software description and used command lines.

sequencing), the total size of the bloom filter and the false positive bifurcation events stored in the file can be approximated by:

$$\underbrace{r \cdot \mathcal{G}}_{\text{Bloom filter}} \quad + \quad \underbrace{3 \cdot 2 \cdot \mathcal{G}\mathcal{D}f^r}_{\text{false bifurcations}} \quad \text{bits}$$

In average, most of the graph nodes are in simple paths, hence 3 possible edges out of each node are likely to produce false bifurcations, and each bifurcation is stored with approximately 2 bits (through the arithmetic coder).

This total size is minimized with :

$$r = \log(\frac{-1}{6 \cdot \mathcal{D} \log f}) / \log f$$

This yields $r = 10.3$ bits for $\mathcal{D} = 50$, very close to the experimentally measured optimal size for the SRR065390 *C.elegans* dataset (70x total coverage $\approx$ 50x kmer coverage for $k = 31$ and read length 100). See figure S4 for experimental measure of the size of bloom filter and

bifurcations on this *C.elegans* dataset.

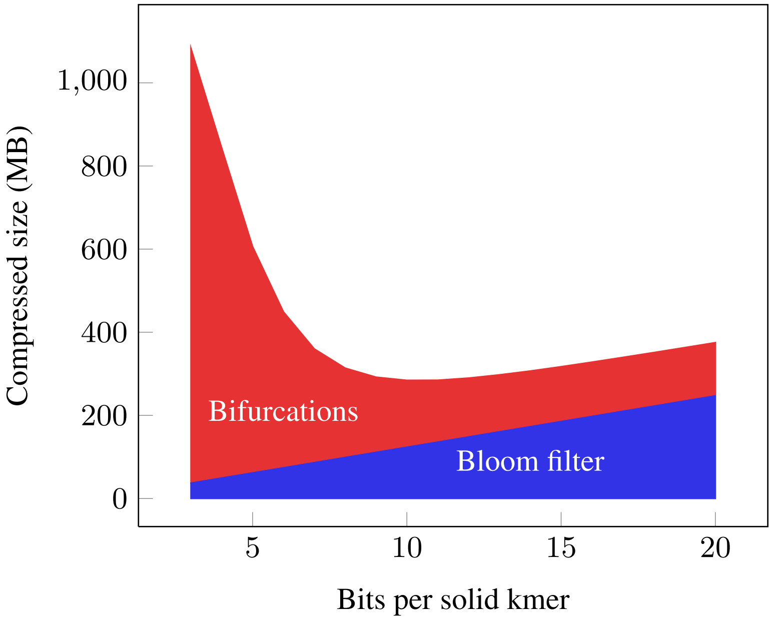

Figure S4: Measured compressed sizes of the bifurcation list and the Bloom filter components with respect to the number of bits per solid kmer parameterized in the Bloom filter. This was obtained for the *C. elegans* 70x WGS dataset.

References

[1] Bonfield, J. K. and Mahoney, M. V. (2013) Compression of FASTQ and SAM format sequencing data.. *PLoS One,* **8**(3), e59190.

[2] Jones, D. C., Ruzzo, W. L., Peng, X., and Katze, M. G. (Dec, 2012) Compression of next-generation sequencing reads aided by highly efficient de novo assembly.. *Nucleic Acids Res,* **40**(22), e171.

[3] Grabowski, S., Deorowicz, S., and Roguski, Ł. (2014) Disk-based compression of data from genome sequencing. *Bioinformatics,* p. btu844.

[4] Deorowicz, S. and Grabowski, S. (2011) Compression of DNA sequence reads in FASTQ format. *Bioinformatics,* **27**(6), 860–862.

[5] P., D. and J.L., G. (1996) Zlib compressed data format specification version 3.3.. *RFC 1950,*.

[6] Hach, F., Numanagic, I., Alkan, C., and Sahinalp, S. C. (Dec, 2012) SCALCE: boosting sequence compression algorithms using locally consistent encoding.. *Bioinformatics,* **28**(23), 3051–3057.

[7] Patro, R. and Kingsford, C. (2015) Data-dependent bucketing improves reference-free compression of sequencing reads. *Bioinformatics,* p. btv248.

[8] Yu, Y. W., Yorukoglu, D., and Berger, B. (2014) Traversing the k-mer Landscape of NGS Read Datasets for Quality Score Sparsification. In *Research in Computational Molecular Biology* Springer pp. 385–399.

[9] Cánovas, R., Moffat, A., and Turpin, A. (2014) Lossy compression of quality scores in genomic data. *Bioinformatics,* **30**(15), 2130–2136.

[10] Kirsch, A. and Mitzenmacher, M. (2006) Less hashing, same performance: Building a better Bloom filter. *AlgorithmsESA 2006,* pp. 456–467.
